# Transcriptomic and metabolomic analyses of cucumber fruit peels reveal a developmental increase in terpenoid glycosides associated with age-related resistance to *Phytophthora capsici*

**DOI:** 10.1038/hortres.2017.22

**Published:** 2017-05-24

**Authors:** Ben N Mansfeld, Marivi Colle, Yunyan Kang, A Daniel Jones, Rebecca Grumet

**Affiliations:** 1 Graduate Program in Plant Breeding, Genetics and Biotechnology, Department of Horticulture, Michigan State University, East Lansing, MI 48824, USA; 2 College of Horticulture, South China Agricultural University, Guangzhou 510642, China; 3 Department of Biochemistry and Molecular Biology, Michigan State University, East Lansing, MI 48824, USA; 4 Department of Chemistry, Michigan State University, East Lansing, MI 48824, USA

## Abstract

The oomycete, *Phytophthora capsici*, infects cucumber (*Cucumis sativus* L.) fruit. An age-related resistance (ARR) to this pathogen was previously observed in fruit of cultivar ‘Vlaspik’ and shown to be associated with the peel. Young fruits are highly susceptible, but develop resistance at ~10–12 days post pollination (dpp). Peels from resistant (16 dpp) versus susceptible (8 dpp) age fruit are enriched with genes associated with defense, and methanolic extracts from resistant age peels inhibit pathogen growth. Here we compared developing fruits from ‘Vlaspik’ with those of ‘Gy14’, a line that does not exhibit ARR. Transcriptomic analysis of peels of the two lines at 8 and 16 dpp identified 80 genes that were developmentally upregulated in resistant ‘Vlaspik’ 16 dpp versus 8 dpp, but not in susceptible ‘Gy14’ at 16 dpp. A large number of these genes are annotated to be associated with defense and/or specialized metabolism, including four putative resistance (R) genes, and numerous genes involved in flavonoid and terpenoid synthesis and decoration. Untargeted metabolomic analysis was performed on extracts from 8 and 16 dpp ‘Vlaspik’ and ‘Gy14’ fruit peels using Ultra-Performance Liquid Chromatography and Quadrupole Time-of-Flight Mass Spectrometry. Multivariate analysis of the metabolomes identified 113 ions uniquely abundant in resistant ‘Vlaspik’ 16 dpp peel extracts. The most abundant compounds in this group had relative mass defects consistent with terpenoid glycosides. Two of the three most abundant ions were annotated as glycosylated nor-terpenoid esters. Together, these analyses reveal potential mechanisms by which ARR to *P. capsici* may be conferred.

## Introduction

Cucumber (*Cucumis sativus* L.) is susceptible to fruit rot caused by the oomycete pathogen, *Phytophthora capsici*.^1^ This soil-borne pathogen can infect and cause severe losses in several crops including members of the Solanaceae and Cucurbitaceae families.^2^ Though *P. capsici* infects vegetative tissues in most crops, in cucumber, the fruits are the primary target of infection.^3^ Thus, a field may appear to be healthy, but the fruits, which are largely located underneath the canopy, will be highly infected and unmarketable. In the field, the primary mode of infection is through the release of motile zoospores by contact with water from rain or irrigation.^1^

While screening germplasm for resistance to this pathogen, an age-related resistance (ARR) was observed.^3,4^ Young fruits of cultivar ‘Vlaspik’ are highly susceptible to infection but become resistant at ~12 days post-pollination (dpp). Cucumber is primarily eaten immature, and thus typically harvested at 8–12 dpp, while fruit ripening and seed maturity is at ~30–35 dpp.^5^ Transcriptome analysis of the early stages of cucumber development, at 0, 4, 8, 12 and 16 dpp revealed two transcriptional shifts that separated these samples into three distinct groups coinciding with three developmental stages: cell division (0–4 dpp), rapid exponential growth (8 dpp) and end of exponential growth (12–16 dpp).^5^ Furthermore, the second transition, at the end of exponential growth, corresponded with the onset of ARR to *P. capsici* and was shown to be enriched for genes associated with biotic and abiotic stress responses.^5^

Developmental, or ontogenic, resistance occurs in a variety of plant–pathogen systems (reviewed by Develey-Rivière and Galiana).^6^ ARR to *P. capsici* has been observed in pepper plants^7^ and in other cucurbits.^4^ ARR may affect the whole plant or be organ-specific.^6^ Fruit-specific ARR, such as observed in cucumber, was also previously shown in other systems. ARR in grape (*Vitis* spp.) berries was observed to black rot,^8^ downy mildew^9^ and powdery mildew (*Uncinula necator*).^10^ ARR of grape berries to powdery mildew was also shown to be genotype-specific.^11^ A recent account of ARR to powdery mildew (*Podosphaera macularis*) was described in hop (*Humulus lupulus*) strobiles.^12^ Strawberry fruit and leaves display ARR to powdery mildew caused by *Podosphaera aphanis*.^13,14^

The molecular mechanisms controlling these ARR traits are not well understood and appear to be highly variable among pathosystems.^6,15^ To study ARR in the *Nicotiana benthamiana*—*P. infestans* pathosystem, a virus-induced gene silencing (VIGS) system was used.^16^ Salicylic acid signaling, independent of NPR1, and ethylene signaling were found to be important in conferring ARR in this system. In addition, production of the sesquiterpenoid phytoalexin capsidiol is controlled by ethylene signaling and is important in ARR to *P. infestans*.^16^ Disease resistance genes (*R* genes) are also implicated in ARR. For example, in the rice—*Xanthomonas oryzae* pv. *Oryza* pathosystem the developmental increase in expression of two genes, *Xa3/Xa26* and *Xa21*, encoding leucine-rich repeat (LRR) receptor kinase-type proteins, are directly linked to ARR.^17,18^

Our recent findings show that ARR of cucumber fruit to *P. capsici* is directly associated with the fruit peel.^19^ Excised peels from post-ARR-aged fruit (15 dpp) placed on susceptible, 8 dpp fruit protected the fruits beneath them.^19^ Furthermore, methanolic extracts from 16 dpp cucumber peels had inhibitory effects on *P. capsici* growth, while those from 8 dpp peels were less inhibitory.^19^ Finally, transcriptome analysis, comparing 8 and 16 dpp fruit peel and pericarp, showed that 16 dpp peels are significantly enriched for genes associated with pathogen defense.

In this work, we sought to identify factors contributing to ARR by examining differential manifestation of ARR in two cucumber cultigens, ‘Vlaspik’ and ‘Gy14’. Both are susceptible to *P. capsici* at 8 dpp; however, at 16 dpp, ‘Gy14’ remains susceptible, while ‘Vlaspik’ becomes resistant. Transcriptomic and untargeted metabolomic analyses were performed on peel samples from 8 and 16 dpp fruit of the two cultigens to identify genes and compounds uniquely upregulated in the resistant ‘Vlaspik’ 16 dpp fruit.

## Materials and methods

### Plant material and RNA extraction

Greenhouse production of pickling cucumber fruit of cultigens ‘Vlaspik’ and ‘Gy14’ was as described in Ando and Grumet.^20^ Flowers were hand pollinated in a staggered manner, such that 8 and 16 dpp fruits were harvested on the same day. For RNA extraction, fruits were peeled using a vegetable peeler and immediately frozen in liquid nitrogen. RNA was extracted using the Trizol procedure, cleaned using RNeasy MinElute Cleanup Kit (Qiagen, Valencia, CA, USA). Samples were DNase (Life Technologies, Inc., Carlsbad, CA, USA) treated for 15 min, and RNA concentration and quality were measured using Qubit 2.0 Fluorometer (Life Technologies, Inc., Carlsbad, CA, USA) and the Agilent 2100 Bioanalyzer (Agilent Technologies, Santa Clara, CA, USA). All samples had a minimum RNA integrity number (RIN) score of 8. For each genotype and age, three biological replicates were prepared; each biological replicate included equal quantities of RNA pooled from two fruits.

### Detached fruit inoculations

Cucumber fruit from cultigens ‘Vlaspik’ and ‘Gy14’ were grown as described above, and pollinated such that 0, 4, 8, 12, 16 and 20 dpp fruits were harvested together. Fruits were processed for a resistance screen as described in Gevens *et al.*^3^ and modified by Colle *et al.*^21^ Briefly, harvested fruits were washed, then surface sterilized by brief immersion in a 2% bleach solution (The Clorox Company, Oakland, CA, USA), rinsed with distilled water several times, and allowed to air-dry. Fruits were placed in incubation trays lined with wet paper towels, to maintain high humidity and covered with clear plastic tops. Zoospore suspensions were prepared from *P. capsisi* isolate OP97^3^ cultured on diluted V8 agar media. After 7 days of culture, the plates were flooded with 6 mL sterile distilled water to release zoospores. A 20 μl aliquot was removed for quantitation by a hemocytometer. The suspension was diluted to a concentration of 1×10^5^ zoospores per mL. Fruits were then inoculated with three, equally spaced, 30 μL droplets of the diluted zoospore suspension. Incubation was performed under constant light at 23–25 °C. Disease progression was observed and ranked on a 1–9 scale as described in Colle *et al.*^21^

### Library preparation, sequencing and differential expression analysis

Illumina TruSeq Stranded mRNA libraries were prepared at the Michigan State University Research Technology Support Facility (RTSF) according to the Illumina protocol (Illumina, San Diego, CA, USA). After quality control and quantitation, all 12 libraries were combined into one pool. This pool was loaded on two lanes of an Illumina HiSeq 2500 Rapid Run flow cell (v1) and sequenced in a 50 nucleotide single end format (SE50) using Illumina Rapid SBS reagents (Illumina, San Diego, CA, USA). Raw reads were deposited in the NCBI Sequence Read Archive (SRA) database under the accession number PRJNA345040.

Reads were cleaned and adaptor sequences were removed using Trimmomatic v. 0.33^22^ and FASTX-Toolkit (http://hannonlab.cshl.edu/fastx_toolkit/index.html). Minimum read length was 36 nt. After quality control with FastQC (http://www.bioinformatics.bbsrc.ac.uk/projects/fastqc) reads were mapped to the ‘Chinese Long’ (v2)^23,24^ cucumber genome using and TopHat v. 1.4.1,^25^ with default settings and an intron size of 10–50 000 nt and no novel junctions. Raw read counts were generated using HTseq^26^ in union and reverse stranded modes. Differential expression (DE) analysis was performed using the R package DEseq2.^27^ Pearson’s correlations of samples were performed on variance-stabilized transformed values, while principal component analysis (PCA) was performed on regularized log values of read counts.

As suggested in the DESeq2 vignette, age and genotype were combined into a single factor for the analysis and contrasts between the four conditions (‘Vlaspik’ 8 dpp, ‘Vlaspik’ 16 dpp, ‘Gy14’ 8 dpp, ‘Gy14’ 16 dpp) were performed. Differentially expressed genes were called significant using an adjusted *P*-value (Benjamini–Hochberg adjustment) and a false discovery rate of <5%. A cutoff expression change of above two-fold was used to define biological significance. Lists of up- and downregulated genes from the contrasts of the conditions were compared. The Venn diagram was created with ggplot2 utilizing the overLapper function (http://faculty.ucr.edu/~tgirke/Documents/R_BioCond/My_R_Scripts/overLapper.R).

### Gene ontology term enrichment analysis

To create an updated Gene Ontology term (GO term) database for cucumber, protein sequences were downloaded from the International Cucurbit Genomics Initiative website (ICuGI, http://www.icugi.org/) and compared by protein BLAST to Arabidopsis TAIR10 proteins (www.arabidopsis.org). GO terms from the best protein hits (*E*-value <1e−10) were extracted. Additional GO terms were extracted from an InterProScan 5 search^28^ of the cucumber protein sequences against all databases. The resulting table of cucumber genes and their matching GO terms is available in Supplementary File 1.

For GO term enrichment analysis, background gene lists were constructed using the MatchIt R package, by Mahalanobis matching, based on the average normalized expression of 5 and 50 genes per DE gene for sets with more and less than a 1000 DE genes, respectively. Using these background gene sets, GO term enrichment analysis was performed with the R package topGO.^29^ Significant terms were defined using the Fisher test, the default ‘weight01’ algorithm, a minimum node size of 6 and *P*-value <0.05 for significance (‘weight01’ *P*-values are considered adjusted). Density plots of log2 normalized counts were compared to ensure relevant background gene set selection. Reduction and visualization of GO terms was performed using REVIGO (http://revigo.irb.hr/)^30^ online tool, using an allowed similarity of 0.5 and the Arabidopsis GO term size database. Results were then transferred to R for plotting.

### Verification by qRT-PCR

The pooled RNA samples used in RNA sequencing (RNA-seq) were also used for oligo (dT)-primed cDNA synthesis using SuperScript II reverse transcriptase according to the manufacturers’ protocol (Life Technologies, Inc.). Samples were then loaded in a 384-well plate for quantitative real-time PCR (qRT-PCR) analysis with an ABI Prism 7900HT (Life Technologies, Inc., Carlsbad, CA, USA) using rEVAlution Master Mix (Syzygy Biotech, Grand Rapids, MI, USA). Primers for genes were designed using NCBI Primer-BLAST (http://www.ncbi.nlm.nih.gov/tools/primer-blast/). Product specificity and reaction efficiencies were verified for each primer pair. A standard curve dilution series (20, 4, 0.8 and 0.16 ng μL^−1^) was made from a pool of 2 μL of each of the cDNA samples. PCR conditions were 95 °C for 2 min then 40 cycles of 95 °C for 5 s, 60 °C for 30 s. Threshold cycle (Ct) levels were translated to cDNA concentration using the relevant standard curve for each gene and normalized to the expression level of *C. sativus Ubiquitin 3* (*CsUBQ3*). Primer pairs used in this analysis are listed in Supplementary File 2.

### Untargeted metabolomic profiling of peel extracts

Methanolic extracts were prepared from cucumber fruit peels of cucumbers grown in two seasons in the greenhouse as described above. Tissues from the first season were those used in the RNA-seq experiment. Approximately, 5 g fresh weight of peel tissue were lyophilized and milled. Once dry, 75 mg of ground peel was extracted for 3 h in 80% HPLC-grade methanol/20% H_2_O at a 1:20 w/v ratio, on a rotary shaker at room temperature. Samples were centrifuged at 15 000 *g* and the supernatant transferred to amber vials. An aliquot from each sample was transferred to a new vial and diluted with Millipore pure water to 25% methanol. A pool of all collected samples was used as a reference for peak alignment.

Chromatographic separations of metabolites were performed using a 7-min gradient using a 2.1×100 mm (1.7 μm) BEH C18 Ultra-Performance Liquid Chromatography (UPLC) column on a Waters Acquity ultra-high pressure LC system (Waters Corp., Milford, MA, USA). A linear solvent gradient of acidified water (0.1% acetic acid, v/v) and acetonitrile as mobile phases A and B, respectively, was used (0 min, 5% B; 0.5 min, 5% B; 5 min, 95% B; 6 min, 95% B; 6.1 min. 5% B; 7 min, 5% B). Column temperature was set at 40 °C and flow rate was 0.4 mL min^−1^. Mass spectra were acquired using negative-ion mode electrospray ionization on a Xevo G2-XS quadrupole time-of-flight mass spectrometer (ESI-QToF-MS) (Waters Corp.), over *m/z* 50–1500 using continuum data acquisition, with mass resolution (*M*/Δ*M*, full-width half maximum) of ~22 000. MS^E^ spectra were acquired in low- and high-energy collision conditions using a collision energy ramp from 15 to 80 V in the latter. Other parameters include capillary voltage of −2.2 kV, desolvation temperature of 350 °C, source temperature of 100 °C, cone gas (N_2_) at 25 L h^−1^, and desolvation gas (N_2_) at 600 L h^−1^. Continuous infusion of the lock mass compound leucine encephalin was performed to allow correction for mass drift.

The fragmentation patterns of ions of interest were further investigated using the same equipment in separate multiplexed collision-induced dissociation (CID) and MS/MS analyses, in both negative and positive ion modes, using a chromatography method scaled to 40 min for greater chromatographic separation. A solvent gradient of 10 mM aqueous ammonium formate (mobile phase A) and acetonitrile (mobile phase B) was used (0 min, 5% B; 2.86 min, 5% B; 28.57 min, 95% B; 34.29 min, 95% B; 34.86 min. 5% B; 40 min, 5% B). Six parallel collision energy functions were used, with centroid data acquisition. Collision cell potentials used in both negative and positive ion fragmentation modes for each function were 6, 15, 30, 45, 60 and 80 V with 0.13 s per function. Other settings were as above.

MassLynx RAW files were imported into the Progenesis QI software (Nonlinear Dynamics, Newcastle upon Tyne, United Kingdom) for pre-processing, peak alignment and picking and abundance normalization. The following ions were grouped into a single measure for each metabolite: [M-H]^−^, [M+Cl]^−^, [M+formic acid-H]^−^, [M+H_2_PO_4_]^−^, [2M+Cl]^−^, [2M+formic acid-H]^−^, [2M+H_2_PO_4_]^−^. Finally, multivariate statistical analysis and PCA were performed using the R statistical software. Chromatograms were trimmed to a retention time range of 0.5–4 min, where most specialized metabolites eluted. Ions with negative absolute mass defects (between 0.7 and 0.97) were filtered from analysis, as these are often inorganic substances. Correlation loadings’ thresholds of 0.4 for age and genotype were used for determining ions of interest. Relative mass defect (RMD) values were calculated as in Ekanayaka *et al.*^31^

## Results

### ARR differentially manifests in the different cucumber cultivars

**Figure 1:**
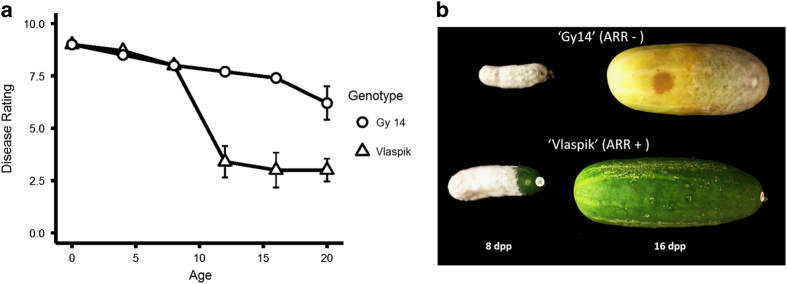
*P. capsici* infection in two cucumber cultigens, ‘Vlaspik’ (ARR+) and ‘Gy14’ (ARR−) in relation to fruit age (days post pollination (dpp)). (**a**) Visual disease ratings. Symptoms were scored on a 9-point scale as per Colle *et al.*^21^ Each point is the mean of five fruits ±s.e.m. (**b**) Cucumber fruit photographed 5 days after inoculation with *P. capsici*.

Not all cucumber cultivars show ARR to *P. capsici*.^32^ For this study, we selected the following two for comparison: ‘Vlaspik’ (ARR+) and ‘Gy14’ (ARR−). As in our previous reports,^3,4^ cv. ‘Vlaspik’ showed a marked decrease in disease ratings from 12 dpp (Figure 1). In contrast, the breeding line ‘Gy14’ remained susceptible to infection with only a slight decrease in disease rating at 20 dpp (Figure 1).

### Age is a key factor affecting gene expression in cucumber peel

RNA-seq analysis was performed on cucumber peels at two ages, 8 and 16 dpp, that is, pre- and post-expression of ARR in ‘Vlaspik’. RNA-seq yielded a total of 334 M pass-filter reads with an average of 26 million clean, trimmed reads per sample (Supplementary File 3). Of these, an ~85% mapped uniquely to the ‘Chinese Long’ (v2)^23,24^ cucumber genome, representing 19 905 of 23 248 genes annotated in the cucumber genome. Pearson’s correlations of biological replicates within treatments were all above 98% (variance-stabilized read counts), indicating good reproducibility (Supplementary Figure 1).

**Figure 2:**
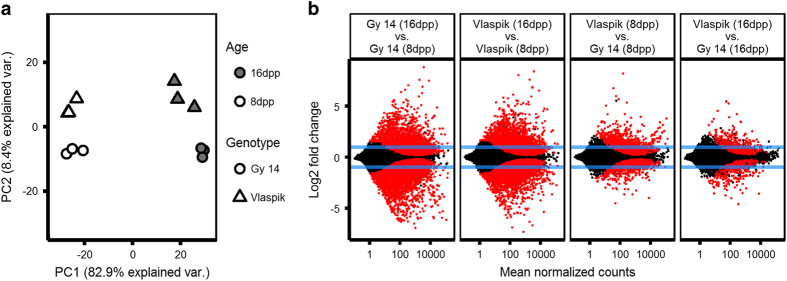
Analysis of transcriptome data from peels of ‘Vlaspik’ (ARR+) and ‘Gy14’ (ARR−) cucumber fruit at 8 and 16 dpp. (**a**) Principal component analysis of the transcriptome data. (**b**) MA plots for pairwise differential expression analysis contrasts between the four conditions. Each point represents a detected gene. Points in red are significantly differentially expressed (adjusted *P*<0.05). Blue lines represent a two-fold change threshold.

**Figure 3:**
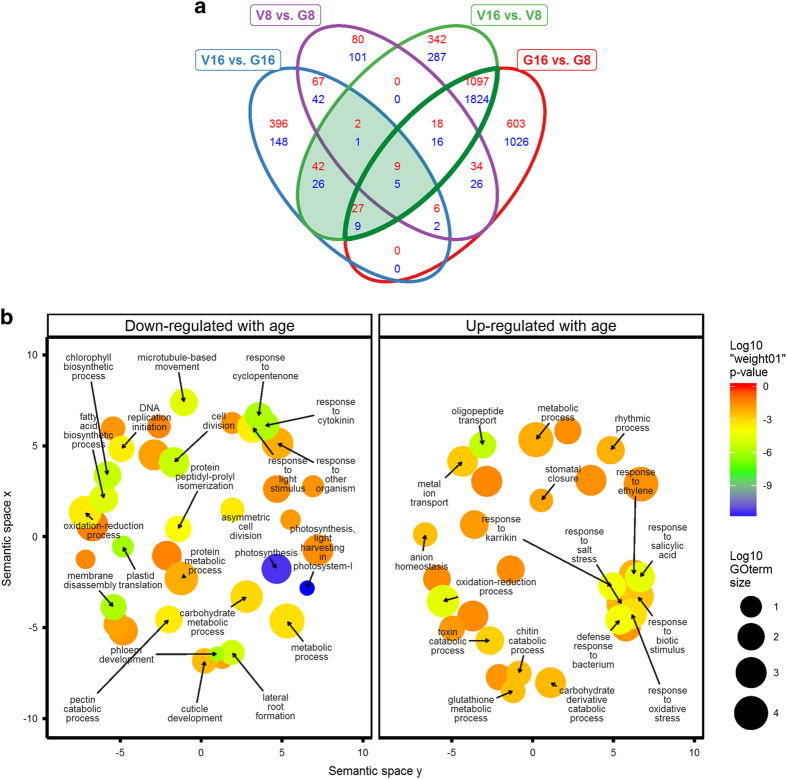
Analysis of differentially expressed genes in ‘Vlaspik’ (ARR+) and ‘Gy14’ (ARR−) cucumber fruit peels at 8 and 16 dpp. (**a**) Venn diagram of all significantly differentially expressed genes (adjusted *P*<0.05, fold change ≥2). Counts in red and blue denote up- and downregulated genes, respectively. The thick green line denotes genes regulated with age in both genotypes. The shaded area denotes genes specifically differentially expressed in resistant ‘Vlaspik’ 16 dpp peels relative to susceptible ‘Vlaspik’ (8 dpp) and 16 dpp ‘Gy14’. V, ‘Vlaspik’; G, ‘Gy14’. (**b**) REVIGO^30^ Visualization of biological process GO term enrichment analysis of age-regulated genes (thick green line above). GO term enrichment was based on topGO analysis using the ‘weight01’ algorithm.^29^ Each circle represents a term with *P*-value<0.05. The proximity of terms represents their semantic similarities and size of the circle represents the size of the term based on Arabidopsis term sizes. The color represents the *P*-value as calculated in topGO. Terms with *P*<0.01 are labeled.

PCA of the RNA-seq data revealed that age was a key factor affecting gene expression in both cultigens, accounting for 82.8% of the observed variance (Figure 2a). A genotype effect was also observed explaining about 8.4% of variance. Pairwise contrasts within age and genotype were performed in DEseq2^27^ and differentially expressed genes were determined based on an adjusted *P*-value <0.05 and a fold change ⩾2 (Figure 2b). Consistent with greater PCA separation based on age, many more genes were differentially expressed in the age comparisons. The two genotypes shared a total of 1151 and 1854 genes that were up- and downregulated with age, respectively (thick line, Figure 3a).

To facilitate GO term enrichment analysis, the cucumber protein sequences were blasted against the Arabidopsis TAIR10 protein database (*E*-value <1e−10) and corresponding GO terms were extracted. Additional GO terms were extracted by running the cucumber protein sequences against all InterProScan 5 databases.^28^ The combined functional assignments are provided in Supplementary File 1. GO term enrichment analysis revealed that the gene set downregulated in 16 dpp peels compared to 8 dpp peels, was enriched primarily for photosynthesis, cell division and growth-related processes, with the top five most significantly enriched terms (*P*-value ⩽1.3 E−6) being: ‘photosynthesis, light harvesting in photosystem I’, ‘photosynthesis’, ‘plastid translation’, ‘phloem development, membrane disassembly’ (Figure 3b). Genes upregulated with age were highly enriched for defense, response to disease, stress-related biological processes, and specialized metabolism. REVIGO analysis^30^ revealed a large cluster of GO terms associated with defense including: ‘response to salicylic acid’, ‘response to ethylene’, ‘response to biotic stimulus’, ‘response to oxidative stress’, ‘defense response to bacterium’, ‘response to salt stress’, ‘response to karrikin’ (Figure 3b). Additional highly enriched terms were ‘oligopeptide transport’ and ‘oxidation–reduction process’.

### Differentially expressed genes associated with ARR

**Figure 4:**
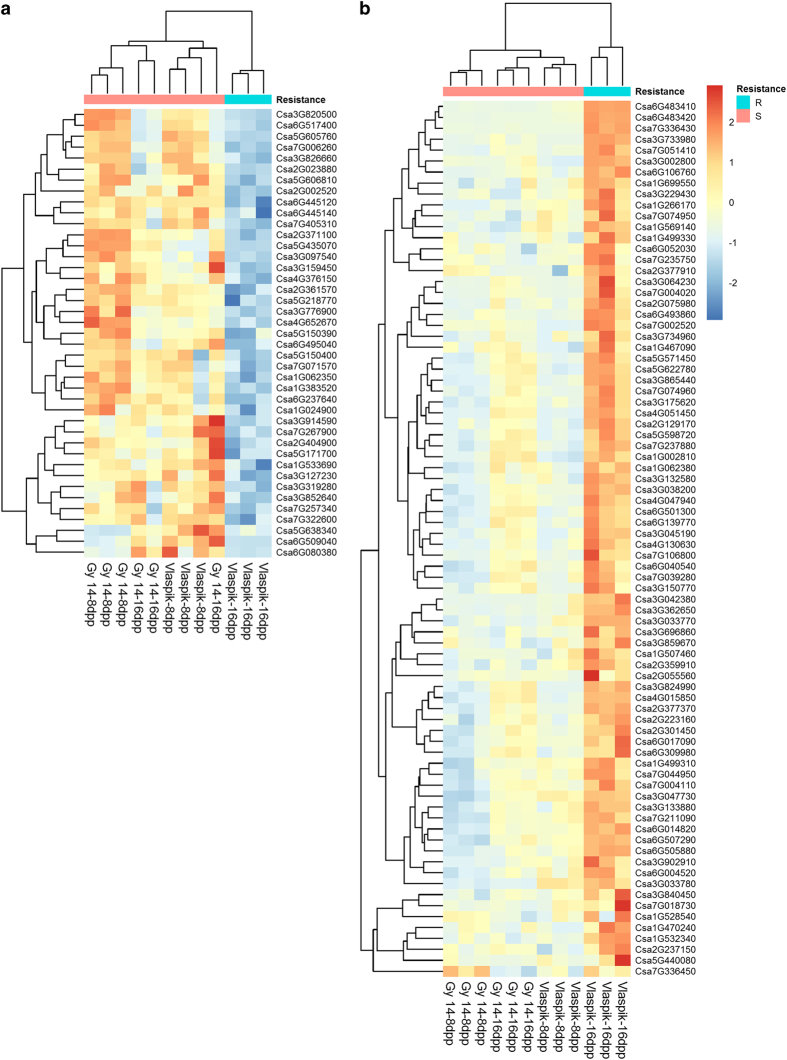
Heatmaps of age-related resistance associated genes. Genes that are downregulated (**a**) or upregulated (**b**) in both resistant ‘Vlaspik’ 16 dpp samples compared to susceptible ‘Gy14’ 16 dpp samples and in resistant ‘Vlaspik’ 16 dpp compared to susceptible ‘Vlaspik’ 8 dpp. Clustering was based on Euclidean distances. Heatmaps are scaled by row. Gene lists are available in Supplementary Files 4 and 5.

To identify genes associated with ARR to *P. capsici* in cucumber, gene expression profiles of the two cultigens were compared at 8 dpp (when both are susceptible) and at 16 dpp (when ‘Vlaspik’ is resistant and ‘Gy14’ is susceptible). Cross listing of all differentially expressed genes defined a subset of genes that were (1) differentially expressed in resistant ‘Vlaspik’ 16 dpp peels compared to susceptible ‘Vlaspik’ 8 dpp peels (age effect); and (2) differentially expressed in the resistant ‘Vlaspik’ 16 dpp peels compared to the susceptible ‘Gy14’ 16 dpp peels (genotype effect) (shaded area Figure 3a, Supplementary Files 4 and 5). This combined analysis of development and genotype allowed us to narrow the >3000 genes differentially expressed with age to ~100. This results in a set of genes that is uniquely up- or downregulated in the resistant fruit (16 dpp ‘Vlaspik’) relative to all susceptible fruits (8 dpp ‘Vlaspik’, 8 dpp ‘Gy14’ and 16 dpp ‘Gy14’). Plotting a heatmap of this set of genes confirmed their specific up- or downregulation in resistant ‘Vlaspik’ 16 dpp samples (Figure 4).

Biological Process (BP) GO term analysis of the upregulated gene set (Table 1A) showed an enrichment of specialized metabolism-associated terms ‘flavonoid biosynthetic process’, ‘isoprenoid biosynthetic process’, ‘oxidation–reduction process’, ‘methylation’, ‘sulfur amino-acid biosynthetic process’, ‘pigment biosynthetic process’ and ‘drug transmembrane transport’ Of the 80 genes defined in this group, 21 were annotated to have function associated with specialized metabolism, including synthesis and decoration of flavonoids and terpenoids. Furthermore, four genes were annotated to be associated with metabolite translocation or vesicle transport. Other genes potentially associated with resistance traits identified within this group included four putative leucine-rich repeat (LRR) receptor kinase-type proteins, a putative WRKY28 transcription factor homolog, as well as four other transcription factors, several calcium receptors and ion transporters. Five genes encoded proteins of unknown function in Arabidopsis and another 12 did not have BLASTP matches with our parameters (E-value<1e−10). Expression patterns of several of the above genes were verified using qRT-PCR (Supplementary Figure 2).

Forty-one genes were downregulated in resistant ‘Vlaspik’ 16 dpp peels compared to both susceptible ‘Vlaspik’ 8 dpp and susceptible ‘Gy14’ 16 dpp peels (Figures 3a and 4). For this set, the top five enriched BP GO terms were ‘defense response, incompatible interaction’, ‘cell wall organization’, ‘response to karrikin’, ‘water transport’, ‘cellular polysaccharide catabolic process’ (Table 1B). The ‘defense response, incompatible interaction’ term resulted from a set of three genes, putative homologs of a chitinase, a lipoxygenase and an aspartyl protease family protein. Furthermore, this set of genes included eight annotated to be involved in specialized metabolism as well. Five genes were annotated to encode extensins, expansins and other cell wall-related proteins. Four genes had no Arabidopsis BLASTP hits, while three encoded unknown proteins.

### Untargeted metabolomic analysis of cucumber peels reveals a dynamic chemical profile

**Figure 5:**
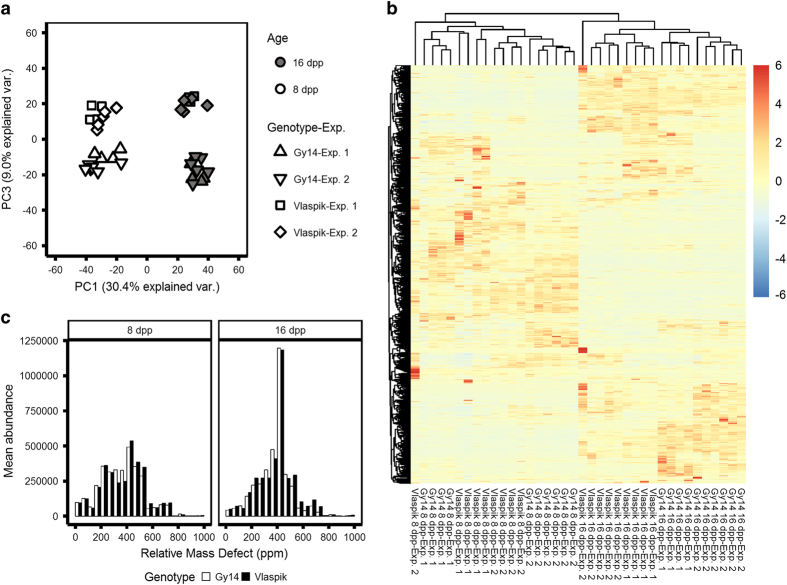
Untargeted metabolomic analysis of methanolic extracts of peel of Vlaspik’ and ‘Gy14’ at 8 and 16 dpp. (**a**) Principal component analysis of ions detected using ultra-performance liquid chromatography negative-ion mode electrospray ionization quadrupole time-of-flight mass spectrometry (UPLC-ESI-QToF-MS). Samples from two greenhouse grown experiments were analyzed. (**b**) Heatmap of the 3289 filtered ion peaks analyzed. Clustering was based on Euclidean distances, and rows are scaled. The full ion list is available in Supplementary File 6. (**c**) Abundance-weighted histograms of all ions binned by relative mass defect (RMD) at 8 and 16 dpp.

Prior observations suggesting inhibition of pathogen growth by methanolic extracts of ‘Vlaspik’ 16 dpp peels^19^ coupled with the above observations that specialized metabolism genes including specific genes in flavonoid and terpenoid synthesis pathways were uniquely up- and downregulated in resistant ‘Vlaspik’ 16 dpp peels, prompted an investigation into the metabolome of the cucumber peel. Metabolomic analyses were performed by untargeted UPLC-ESI-QToF-MS on methanolic peel extracts of the cucumber cultigens. After filtering, a total of 3289 ion peaks were observed in the set of peel extracts (Supplementary File 6). PCA of the metabolome data showed tight clustering of replicate samples both within experiments and between experiments grown at different times in the greenhouse, confirming reproducibility of the results (Figure 5a). PCA further revealed that, similar to the transcriptome data, age is a strong factor influencing the metabolome of the cucumber fruit peel, explaining 30.4% of the observed variance, represented by principal component (PC) 1. The variance attributed to genotypic separation of samples was explained by PC3 (9% explained variance). Heatmap visualization of all the analyzed metabolites (Figure 5b) showed distinct hierarchical clustering of the samples by age. Notably, the great majority of ions showed age-related changes in abundance, either increasing or decreasing with age.

In order to assess the classes of these ions, RMD values were calculated and their distributions were plotted in abundance-weighted histograms (Figure 5c). RMD values, calculated in parts per million (ppm) as (mass defect/measured monoisotopic mass)×10^6^, serve as a measure of the fractional hydrogen content of a detected ion, thus assisting in classification of the ion based on the metabolite’s biosynthetic origin.^31^ The majority of ions detected, from both the 8 and 16 dpp peel extracts, ranged in values from 0 to ~750 ppm. As expected, lipophilic ions, with RMD >750 ppm were largely excluded from the 80% methanolic extracts. While profiles of both 8 and 16 dpp extracts included similar ranges of ions, a strikingly high peak of ions with RMD corresponding with terpenoid glycosides (400–450 ppm) was observed in extracts from 16 dpp peels.

### Compounds uniquely abundant in resistant ‘Vlaspik’ 16 dpp peels

**Figure 6:**
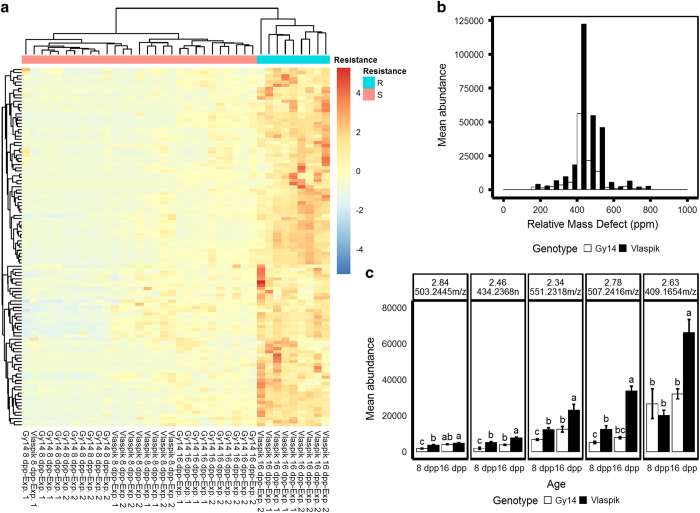
Ions specifically associated with methanolic peel extracts from resistant ‘Vlaspik’ fruit. (**a**) Heatmap of 113 ions detected as uniquely abundant in resistant peels. Ions shown are positively correlated with both PC1 and PC3 (Figure 5a) (correlation loadings >0.4). Clustering was based on Euclidean distances, and rows are scaled. (**b**) Abundance-weighted histogram of these ions, comparing ‘Vlaspik’ and ‘Gy14’ at 16 dpp. (**c**) The top five most abundantly detected ions within this group, plotted by age and genotype (Tukey’s honest significant difference, *α*=0.05).

A multivariate analysis approach was used to further elucidate the difference between samples, and identify ions uniquely abundant in 16 dpp ‘Vlaspik’ peels. Correlations between the normalized abundance data for each ion and each principal component were calculated (correlation loadings). Based on the group separations in PCA, ions strongly positively correlated (correlation loadings >0.4) to both PC1 and PC3 would be uniquely abundant in resistant ‘Vlaspik’ 16 dpp extracts and may thus be associated with ARR. A total of 113 ions matched these criteria, and a heatmap confirmed their increased abundance in ‘Vlaspik’ 16 dpp peel extracts (Figure 6a). Among the ions uniquely abundant in 16 dpp ‘Vlaspik’ peels relative to 16 dpp ‘Gy14’ peels, was a distinct peak of compounds with RMD values of 400–450 ppm, consistent with terpenoid glycosides (Figure 6b).

The most abundant compounds in this set were further analyzed (Figure 6c). Among the top five most abundant were three distinctly abundant ions, *m/z* 409.16, *m/z* 507.24 and *m/z* 551.23, with relative abundance levels of more than 20 000—more than two-fold higher than any of the other ions in this group. All three ions had RMDs consistent with terpenoid glycosides. An additional ion, *m/z* 463.24, had the same retention time as *m/z* 507.24 (2.78 min) and the mass difference between them correspond to a 44 Da loss of CO_2_. Therefore, the abundance levels presented for *m/z* 507.24 represent the sum of both *m/z* 463.24 and *m/z* 507.24.

### Annotation of terpenoid glycosides abundant in peels of resistant cucumbers

Negative mode multiplexed CID mass spectra of the most highly abundant compound detected in this set, *m/z* 409.17 (RMD=418 ppm) yielded only a very small fragment at *m/z* 263.14, due to a neutral loss of 146.02 Da. Positive mode tandem MS analysis (Supplementary Figure 3) of the ammonium adduct at *m/z* 428.21 yielded fragment ions at *m/z* 163.06, 145.04, 127.03 and 109.02 corresponding to the fragmentation of a rhamnose. The neutral loss of 265.14 Da observed in ESI+ mode is in agreement with the fragment ion detected at *m/z* 263.14 observed in the negative mode CID spectra. As there were no other relevant fragmentation products detected, the aglycone was annotated with the proposed formula C_12_H_23_O_6_^−^ (−4.6 ppm error).

The MS/MS spectrum (Supplementary Figure 4) of products of *m/z* 507.24 (RMD=476 ppm), the second most abundant ion, revealed a major fragment at *m/z* 463.24 (RMD=536 ppm) and *m/z* 421.23 (RMD=565 ppm), corresponding to a loss of CO_2_ and C_2_H_2_O, characteristic of a malonate ester.^31^ Further fragmentation yielded ions at *m/z* 403.22 (RMD=569 ppm) concordant with a loss of H_2_O, followed by a neutral loss of 162 Da, corresponding to a loss of glucose. The final fragment, at *m/z* 241.1764 (RMD=731 ppm) had the proposed formula of C_14_H_25_O_3_^−^ and thus annotated as a nor-sesquiterpene. As expected with a terpenoid glycoside, the RMD generally increased as fragment ion mass decreased. Based on the fragmentation characteristics, the compound can be annotated as a nor-sesquiterpenoid glycoside malonate ester.

Multiplexed CID mass spectra (Supplementary Figure 5) of the metabolite yielding the third most abundant ion, *m/z* 551.23 (RMD=420 ppm), were efficient in revealing informative fragments. The most abundant fragment ion at *m/z* 243.12 (RMD=520 ppm), corresponded to a loss of rutinose (308 Da). This was followed by a small fragment at *m/z* 225.11 (RMD=470 ppm), corresponding to a loss of H_2_O. The spectra showed fragments, characteristic of acetate esters, formed by the losses of C_2_H_4_O_2_ and CO_2_ to give *m/z* 183.10 (RMD=563 ppm) and 139.11 (RMD=827 ppm), respectively. Again, the characteristic increase in RMD alongside the reduction in fragment mass was observed. As it did not undergo further fragmentation, the fragment ion at *m/z* 139.1151 was annotated as a nor-monoterpene core (C_9_H_15_O^−^), and thus the original compound could be annotated as a nor-monoterpenoid diglycoside acetate ester.

## Discussion

### Transcriptional reprogramming toward defense in post-exponential growth stage peels

RNA-seq was performed on cucumber peels at two distinct stages of fruit development, 8 and 16 dpp, during and after exponential growth, respectively. PCA of the transcriptome data showed that age was a major factor differentiating the samples, explaining 82.4% of the observed variance between samples (Figure 2a). ‘Defense response’ was the most enriched GO term in genes upregulated with age in both cucumber cultigens, while the majority of downregulated genes were associated with photosynthesis and growth. We thus confirm our previous observations^5,19^ of a transcriptomic shift away from growth toward defense, at the end of exponential stage of fruit growth, perhaps mirroring competition between growth and defense, frequently observed in whole-plant systems.^33,34^ This developmental period, though not thoroughly studied in the fruit development literature, may be a critical stage in which the fruit and its peel in particular, play a crucial role in protecting the metabolic investment the plant has made in fruit and seed maturation prior to dispersal.

### ARR-associated genes

To identify genes specifically associated with ARR, we compared the transcriptomes of peel tissue of ‘Vlaspik’, a cultivar that expresses ARR at 16 dpp, and ‘Gy14,’ a breeding line that remains susceptible throughout fruit development (Figure 1). By comparing cultivars that do and do not exhibit ARR, we were able to use a combination of factors (age and genotype) to distinguish general developmental changes from those specifically associated with ARR. Thus, of the ~3000 genes (up- and downregulated) that were differentially expressed with development in both cultivars, only 120 transcripts were uniquely differentially expressed in the cultivar exhibiting ARR at the resistant age. Several of the 80 genes identified to be specifically upregulated in 16 dpp ‘Vlaspik’ are putative R genes or signaling factors that may have functions in conferring resistance.

The manifestation of ARR may be attributable to either preformed or induced resistance mechanisms; both cases would require developmental changes that allow for age-regulated expression of the defense mechanism. Preformed responses may include chemical factors capable of inhibiting pathogen growth, as suggested by inhibitory effects of methanolic peel extracts.^19^ Induced responses may include developmentally regulated expression of receptors, signal transduction genes or their respective regulators. In tomato, the LRR gene *Cf9-B* conferring mild resistance to *Cladosporium fulvum* is genetically linked to ARR.^35^ In rice, increased expression of LRR genes, *Xa3/Xa26* and *Xa21,* conferring ARR to bacterial blight was shown to be developmentally regulated.^17,18^ It is also possible that a combination of mechanisms contributes to resistance. For example, R genes may contribute to resistance alongside performed metabolites, or pathogen recognition by R genes may modulate further accumulation or modification of specific anti-microbial metabolites.^36^

Among the upregulated genes detected in our analysis, were four genes encoding putative LRR-receptor-like proteins (*Csa3G229430, Csa4G015850, Csa5G168890 *and* Csa4G051450*). Interestingly, all four of these cucumber fruit putative LRR genes were shown to be differentially regulated in leaves in response to *Pseudoperonospora cubensis*, another oomycete pathogen, and the causal agent of downy mildew in cucurbits.^37^ One of these genes (*Csa3G229430*) is a putative homolog of the tomato receptor for Ethylene-Inducing Xylanase (EIX), a fungal elicitor of plant defense response.^38^ This gene also appears to be developmentally regulated in tomato fruit, as it has previously been shown to be upregulated in response to *Botrytis cinerea* infection uniquely in mature green tomato fruit but not in ripe fruit.^39^

Several other genes uniquely upregulated in resistant ‘Vlaspik’ 16 dpp that may function in resistance include genes associated with transport of calcium and other ions (*Csa3G045190, Csa2G359910, Csa1G532340*), as well as a calcium dependent protein kinase (CPK) homolog, *Csa6G052030*. Calcium homeostasis and transport act in pathogen-associated molecular pattern (PAMP) signaling and response by initiating reactive oxygen species (ROS) bursts as well as inducing expression of immunity genes.^40,41^ An upregulation of a *WRKY28* transcription factor homolog (*Csa6G139770*) was also observed. In *Arabidopsis*, WRKY28 directly binds to the promoter and regulates the key SA biosynthesis gene *isochorismate synthase (ICS)*.^42^ It was further shown that among other WRKY TFs, WRKY28 is phosphorylated by CPKs, thus facilitating binding to target gene promoters.^43^ Furthermore, overexpression of *WRKY28*, in *Arabidopsis* led to increased resistance to the necrotrophic pathogen, *Sclerotinia sclerotiorum*.^44^ Thus, increased expression of these genes in resistant ‘Vlaspik’ 16 dpp peels could function as a developmentally regulated mechanism in ARR to *P. capsici*.

### The cucumber peel metabolome is developmentally plastic

Enrichment of specialized metabolism genes in the transcriptome of resistant 16 dpp ‘Vlaspik’ peels, and our previous study showing that methanolic extracts from 16 dpp peels had inhibitory effects on *P. capsici* growth,^19^ suggested that metabolic changes in the peel may contribute to ARR to *P. capsici*. Given the multiple roles that dermal tissues play in interfacing with the abiotic and biotic environment, fruit peel is highly metabolically diverse and can contain may defensive compounds.^45^ As the specific compounds that may be involved in ARR are not known, we performed untargeted metabolic profiling, an approach that has only recently begun to be applied to study fruit peels, particularly in apple.^46,47^ Untargeted metabolomic profiling of the methanolic peel extracts from cucumber showed that the cucumber peel metabolome is highly plastic, undergoing extensive change with development. Virtually every ion detected had some degree of difference in accumulation with age. A clear increase in ions with RMD values consistent with terpenoids and their glycosides was observed in peel extracts of 16 dpp fruit, regardless of genotype.

### Terpenoid glycosides are the most abundant compounds uniquely detected in resistant peels

Of the 80 uniquely upregulated genes in resistant ‘Vlaspik’ 16 dpp fruit, 21 genes were associated with synthesis and decoration of specialized metabolites, including flavonoids and terpenoids. Genes annotated to function specifically in flavonoid metabolism included: a flavanone 3-hydroxylase (*Csa3G002800*), flavonol synthase (*Csa6G040540*) and isoflavone reductases (*Csa7G002520, Csa7G004020*). Despite increased expression of these putative flavonoid synthesis and modification genes in ‘Vlaspik’ 16 dpp peels, our RMD-based analysis of the extracts, only revealed a slight increase in unique accumulation of ions potentially in this class. Furthermore, no previously known flavonoids were identified as uniquely abundant in these extracts. Possible explanations for the lack of correspondence between gene expression and metabolite accumulation maybe due to their neo-functionalization for production/modification of unknown polyphenolics or other compounds. Alternatively, these genes may be otherwise subject to post-transcriptional repression. One example in melon peel showed that accumulation of the flavonoid pigment, naringenin-chalcone, was controlled post-transcriptionally by an F-BOX protein.^48^ The incorporation of flavonoids into the cell wall or cuticle matrices, might also minimize their extraction using our methods.^49,50^

Multivariate and RMD analyses of the metabolomics profiles showed a general enrichment of terpenoids in 16-day-old peels, and a unique enrichment of terpenoid glycosides in extracts of resistant ‘Vlaspik’ 16 dpp peels (Figure 6a). Two of the three ions can be annotated as malonated and acetylated nor-terpenoid glycosides. With the exception of cucurbitacins, little has been reported about synthesis or accumulation of soluble terpenoids or nor-terpenoids and their respective glycosides in cucumber. The cucurbitacins, bitter tetracyclic triterpenes, are perhaps the most infamous compounds in the cucurbit family.^51,52^ Due to their function in defense from herbivory, insects and pathogens, their medicinal value and their association with cucurbit domestication, these compounds have been studied thoroughly and, recently, the genetic mechanisms involved in their production were elucidated.^52,53^ There are, however, also reports of volatile terpenoid production in cucumber. For example, following herbivory by spider-mites, cucumber leaves release a blend of volatile terpenoids that attract predatory mites, natural enemies of the herbivores.^54–56^

Consistent with the detection of uniquely abundant nor-terpenoid glycosides, was the specific upregulation of expression of terpenoid synthesis genes in resistant ‘Vlaspik’ 16 dpp peels. *Csa3G038200* encodes a putative hydroxymethylglutaryl-CoA synthase, the second enzyme in the mevalonate pathway of isoprenoid synthesis; *Csa3G042380* encodes a putative terpene synthase/cyclase family protein (TPS); and *Csa7G211090* encodes a putative small subunit of the heteromeric geranyl(geranyl) diphosphate synthase (G(G)PP-SSU). Recently, transcriptomic, metabolomic and functional analyses of terpene synthesis genes, including those upregulated in our data, were performed in several cucumber tissues.^57^ That study confirmed the functions of several genes in volatile terpene production. For example, Csa7G211090, was demonstrated to function as an G(G)PP-SSU enzyme and is able to produce geranyl pyrophosphate (GPP) for monoterpene production. The terpenoid skeleton synthesis enzymes detected in our analysis, along with a putative malonyltransferase and acyltransferase (*Csa1G499310, Csa1G499330*), a UDP glycosyltransferase (*Csa7G051410*) and a putative CYP450 gene (*Csa6G501300*), could function in synthesis and decoration of the identified compounds and perhaps in conferring ARR, yet further functional evidence is necessary to determine whether this is the case.

An additional group of genes that may function in modification of these or other specialized metabolites was also upregulated in resistant ‘Vlaspik’ 16 dpp peels. This set included putative homologs of a glycosyl hydrolase (*Csa1G528540*), flavine-containing monooxygenases (*Csa3G033770, Csa3G033780*), *O*-methyltransferases (*Csa3G047730, Csa7G018730, Csa7G039280*) and other oxidoreductases (*Csa7G074950, Csa7G074960*). An upregulation of genes associated with vesicle or metabolite transport (*Csa1G507460, Csa2G129170, Csa5G598720, Csa5G622780*), was also observed, perhaps in relation to vacuole sequestration or secretion of specialized metabolites.

## Conclusions

The phenomenon of ARR has been observed in several pathosystems, however, the molecular mechanisms regulating this trait are poorly understood. We have shown that fruit of the cultivar ‘Vlaspik’ develop resistance to *P. capsici* as they complete exponential growth, while those of ‘Gy14’ remain susceptible. Transcriptomic and metabolomic comparisons of fruit peel from these two cucumber lines identified several factors potentially contributing to ARR to this pathogen. These included increased expression of several LRR type genes, transcription factors, calcium-related genes and many specialized metabolism genes in both terpene and flavonoid synthesis pathways, as well as an increased abundance of terpenoid glycosides in resistant ‘Vlaspik’ 16 dpp peels.

## Figures and Tables

**Table 1:** GO term enrichment of differentially expressed genes in resistant ‘Vlaspik’ 16 dpp peels

*GO ID*	*Name*	*Ontology*	*‘weight01’* *P-value (<0.05)*
*A. Genes uniquely upregulated in resistant ‘Vlaspik’ 16 dpp peels*			
GO:0009813	Flavonoid biosynthetic process	Biol. Proc.	0.0014
GO:0008299	Isoprenoid biosynthetic process	Biol. Proc.	0.0023
GO:0055114	Oxidation–reduction process	Biol. Proc.	0.0023
GO:0032259	Methylation	Biol. Proc.	0.0027
GO:0000097	Sulfur amino-acid biosynthetic process	Biol. Proc.	0.0057
GO:0046148	Pigment biosynthetic process	Biol. Proc.	0.0449
GO:0006855	Drug transmembrane transport	Biol. Proc.	0.0499
GO:0008757	*S*-adenosylmethionine-dependent methyltransferase activity	Mol. Func.	0.00042
GO:0016706	Oxidoreductase activity, acting on paired donors, with incorporation or reduction of molecular oxygen, 2-oxoglutarate as one donor, and incorporation of one atom each of oxygen into both donors	Mol. Func.	0.0008
GO:0031418	l-ascorbic acid binding	Mol. Func.	0.00558
GO:0050660	Flavin adenine dinucleotide binding	Mol. Func.	0.00583
GO:0050661	NADP binding	Mol. Func.	0.01014
GO:0046872	Metal ion binding	Mol. Func.	0.01704
GO:0015297	Antiporter activity	Mol. Func.	0.02589
GO:0016709	Oxidoreductase activity, acting on paired donors, with incorporation or reduction of molecular oxygen, NAD(P)H as one donor, and incorporation of one atom of oxygen	Mol. Func.	0.03052
GO:0015238	Drug transmembrane transporter activity	Mol. Func.	0.03052
GO:0016667	Oxidoreductase activity, acting on a sulfur group of donors	Mol. Func.	0.03389
			
*B. Genes uniquely downregulated in resistant ‘Vlaspik’ 16 dpp peels*			
GO:0009814	Defense response, incompatible interaction	Biol. Proc.	0.0016
GO:0071555	Cell wall organization	Biol. Proc.	0.0030
GO:0080167	Response to karrikin	Biol. Proc.	0.0038
GO:0006833	Water transport	Biol. Proc.	0.0059
GO:0044247	Cellular polysaccharide catabolic process.	Biol. Proc.	0.0059
GO:0006865	Amino-acid transport	Biol. Proc.	0.0059
GO:0009740	Gibberellic acid-mediated signaling pathway	Biol. Proc.	0.0204
GO:0009658	Chloroplast organization	Biol. Proc.	0.0241
GO:0072593	Reactive oxygen species metabolic process	Biol. Proc.	0.0324
GO:0044802	Single-organism membrane organization	Biol. Proc.	0.0369
GO:0051649	Establishment of localization in cell	Biol. Proc.	0.0407
GO:0030243	Cellulose metabolic process	Biol. Proc.	0.0466
GO:0015171	Amino-acid transmembrane transporter activity	Mol. Func.	0.0055
GO:0015250	Water channel activity	Mol. Func.	0.0055
GO:0015238	Drug transmembrane transporter activity	Mol. Func.	0.0127
GO:0000287	Magnesium ion binding	Mol. Func.	0.0224
GO:0004553	Hydrolase activity, hydrolyzing *O*-glycosyl compounds	Mol. Func.	0.0262
GO:0051119	Sugar transmembrane transporter activity	Mol. Func.	0.0385
