# Supplementary material for: Transcriptomic and metabolomic analyses of cucumber fruit peels reveal a developmental increase in terpenoid glycosides associated with age-related resistance to Phytophthora capsici
Source: Hortic Res. 2017 May 24;4:17022–. doi: 10.1038/hortres.2017.22 (PMC5442961; doi:10.1038/hortres.2017.22)
Supplement: Supplementary File 6 [file hortres201722-s7.docx]

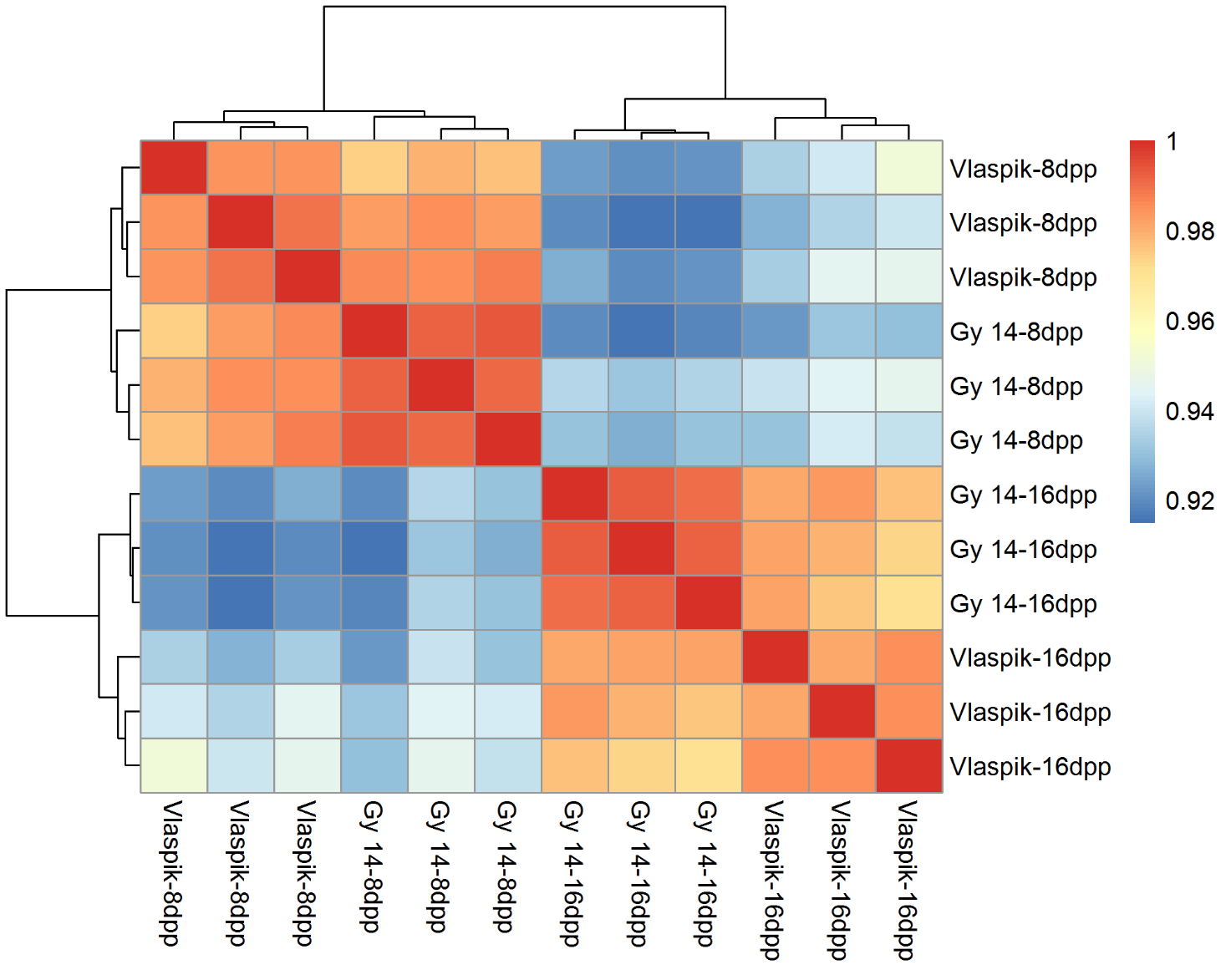


**Supplementary Figure 1.** A heatmap of Pearson’s correlations of variance stabilized read counts from peels of ‘Vlaspik’ and ‘Gy 14’ cucumber fruit at 8 and 16 days post-pollination (dpp).

**
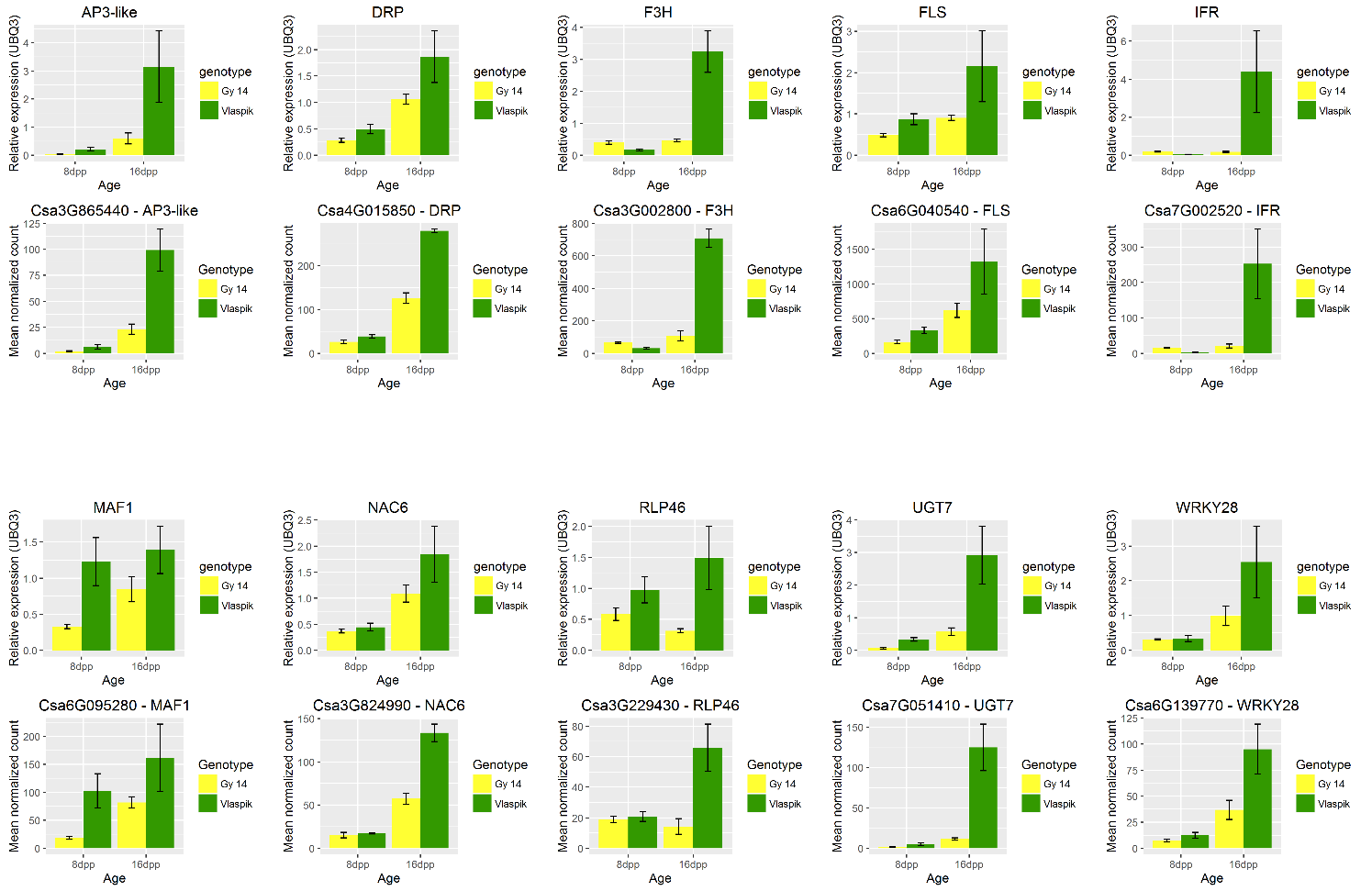
**

**Supplementary Figure 2.** Validation of RNAseq gene expression using quantitative real time PCR (qRT-PCR). In each of the two pairs of rows, the top row represents qRT-PCR results (Relative expression vs. *CsUBQ3*) and the bottom row represents RNAseq normalized read counts. The annotations are based on Arabidopsis best hits. AP3-like: APETALA 3 DNA binding / transcription factor; DRP: disease resistance protein (NBS-LRR class), putative; F3H: FLAVANONE 3-HYDROXYLASE; FLS FLAVONOL SYNTHASE; IFR: ISOFLAVONE REDUCTASE, putative; MAF1: MADS AFFECTING FLOWERING 1 transcription factor; NAC6: ARABIDOPSIS NAC DOMAIN CONTAINING PROTEIN 6 protein heterodimerization/ protein homodimerization/ transcription factor; RLP46: Receptor Like Protein 46 kinase/ protein binding; UGT7: UDP-GLUCOSYLTRANSFERASE 74F2; WRKY28: WRKY28 transcription factor. Error bars are +/- SEM.


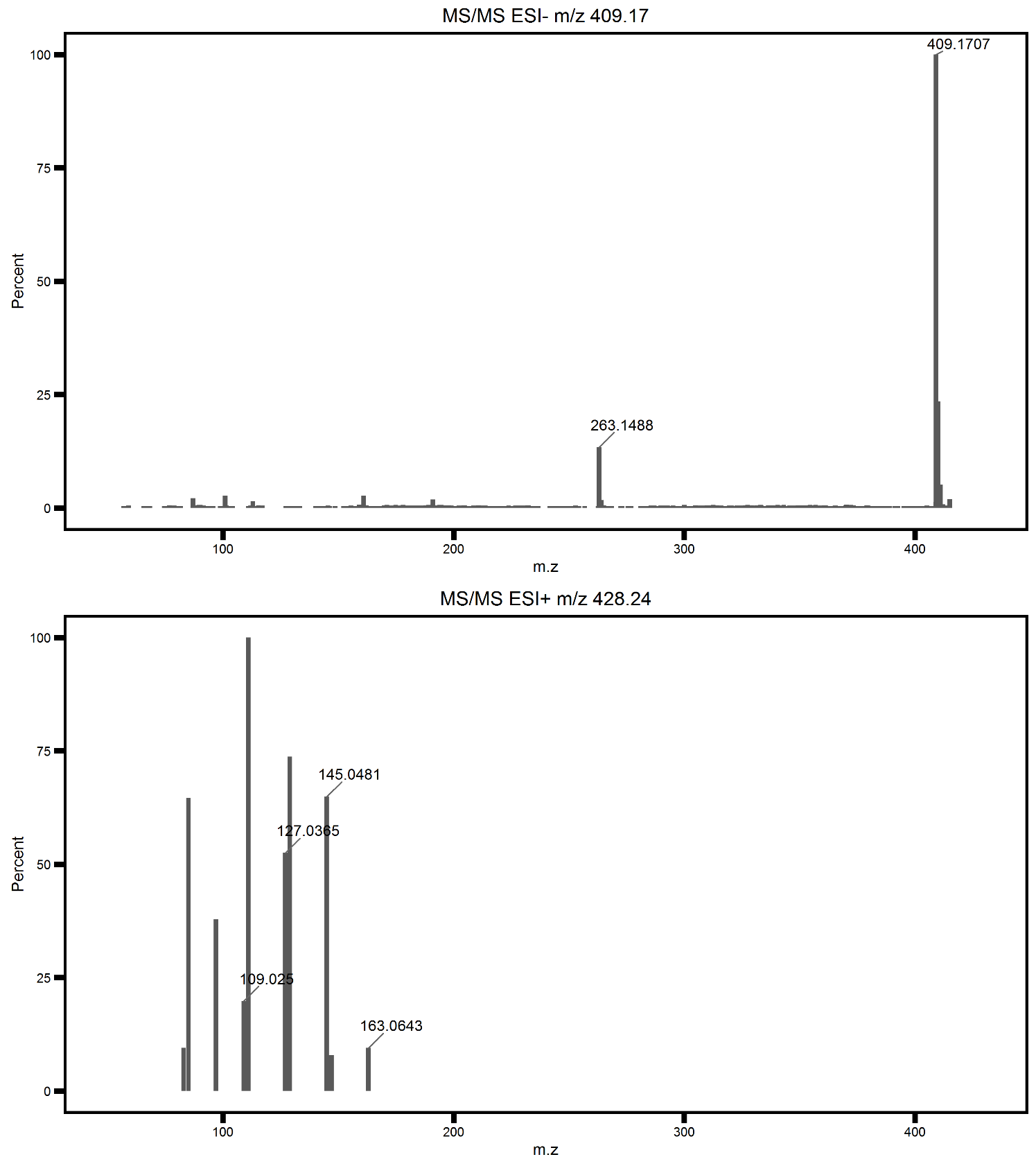


**Supplementary Figure 3.** Negative and positive ion mode product ion MS/MS spectra of products from [M-H]^-^ (*m/z* 409.17) and [M+Na]^+^ (*m/z* 428.24), respectively (Most abundant compound). The lower spectrum displays the positive ion mode.


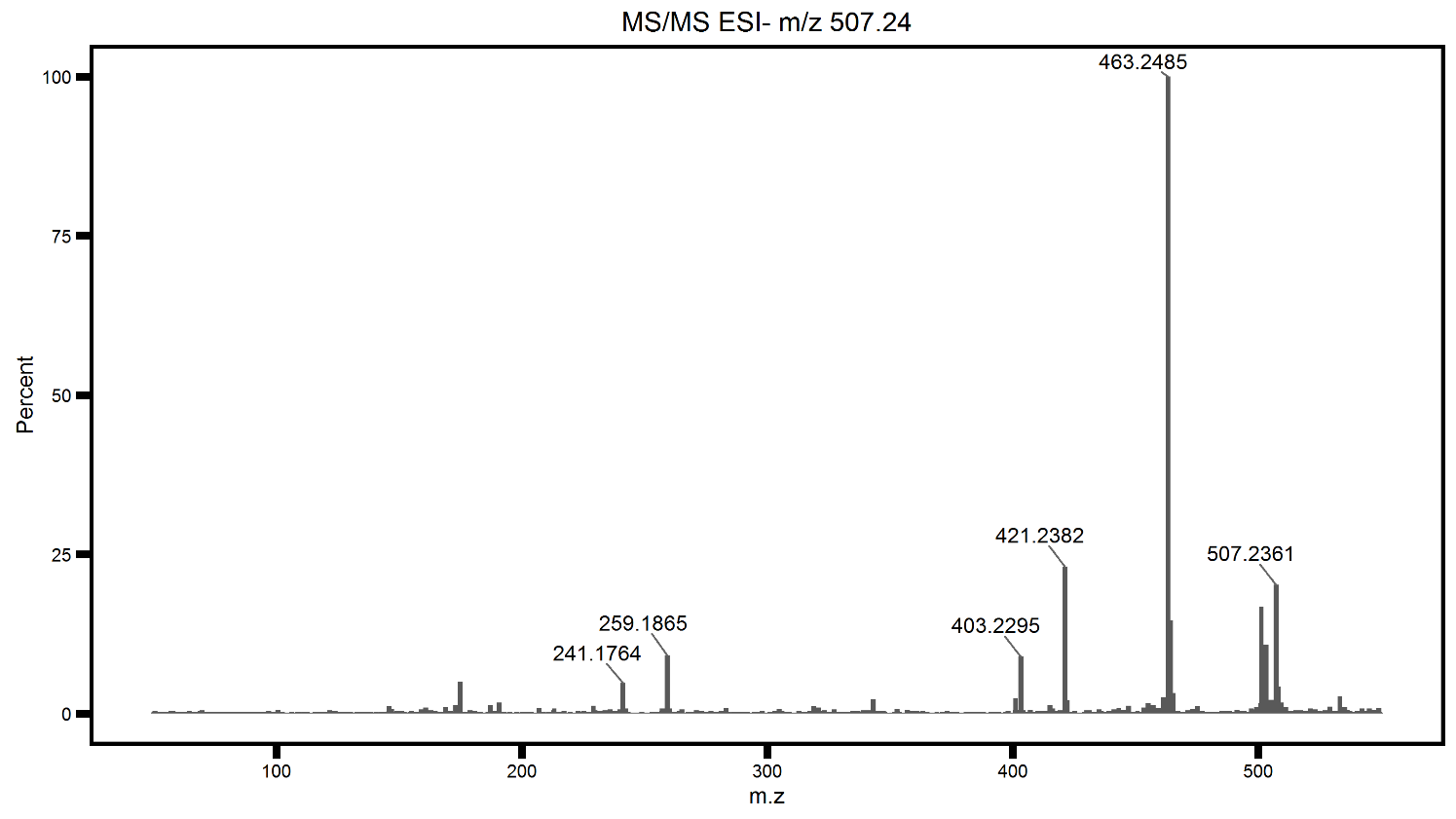


**Supplementary Figure 4.** Negative ion mode product ion MS/MS spectrum of products from [M-H]^-^ (*m/z* 507.24) for nor-sesquiterpenoid glycoside malonate ester (2^nd^ most abundant compound).


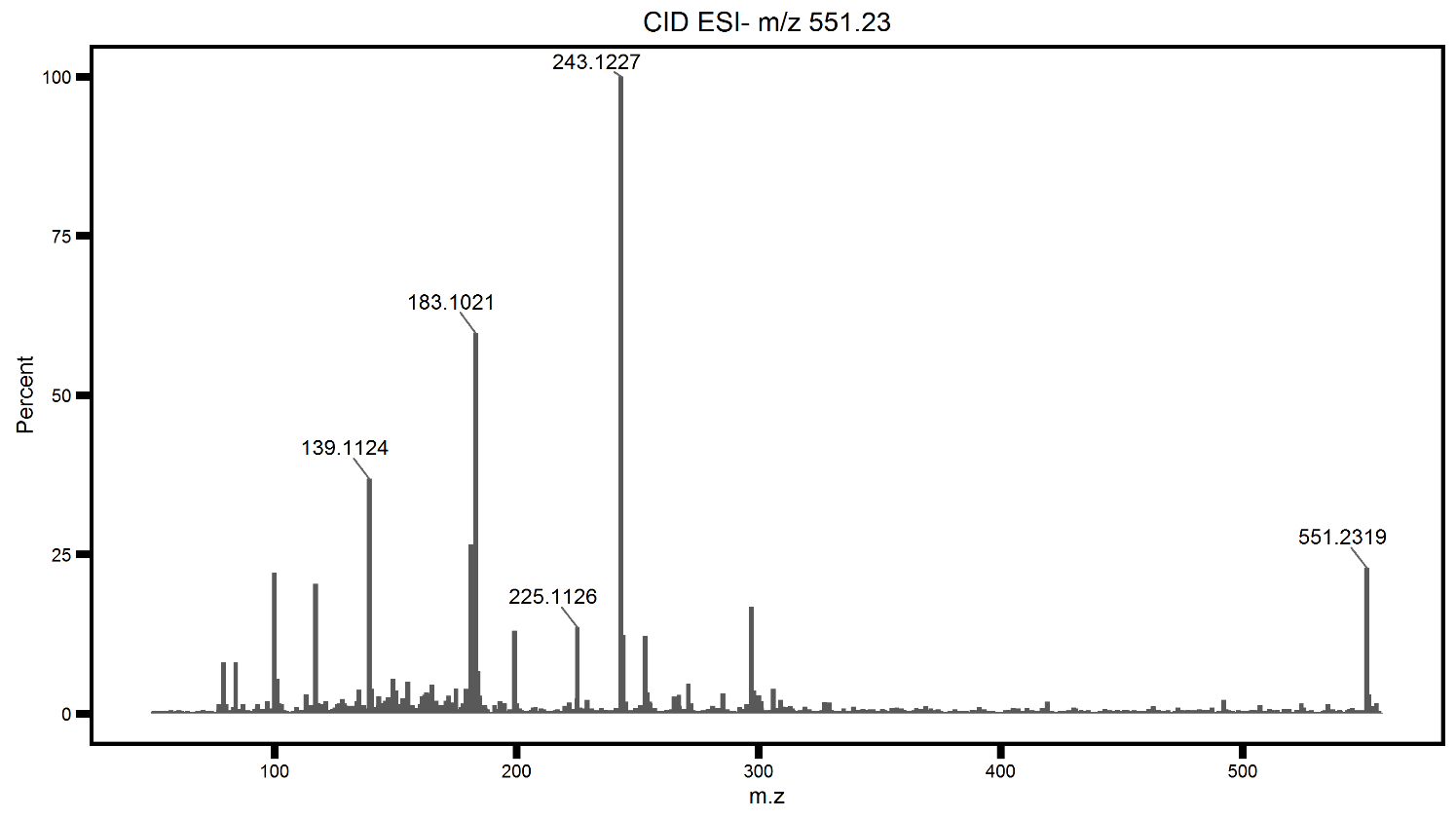


**Supplementary Figure 5.** Negative ion mode multiplexed CID mass spectrum of nor-monoterpenoid diglycoside acetate ester (*m/z* 551.23 is [M-H]^-^) (3^nd^ most abundant compound).
